# Generation of Brain Microvascular Endothelial-Like Cells from Human Induced Pluripotent Stem Cells by Co-Culture with C6 Glioma Cells

**DOI:** 10.1371/journal.pone.0128890

**Published:** 2015-06-10

**Authors:** Haruka Minami, Katsuhisa Tashiro, Atsumasa Okada, Nobue Hirata, Tomoko Yamaguchi, Kazuo Takayama, Hiroyuki Mizuguchi, Kenji Kawabata

**Affiliations:** 1 Laboratory of Stem Cell Regulation, National Institute of Biomedical Innovation, 7-6-8, Saito-Asagi, Ibaraki, Osaka 567–0085, Japan; 2 Laboratory of Biomedical Innovation, Graduate School of Pharmaceutical Sciences, Osaka University, 1–6 Yamadaoka, Suita, Osaka 565–0871, Japan; 3 Laboratory of Hepatocyte Regulation, National Institute of Biomedical Innovation, 7-6-8, Saito-Asagi, Ibaraki, Osaka 567–0085, Japan; 4 Laboratory of Biochemistry and Molecular Biology, Graduate School of Pharmaceutical Sciences, Osaka University, 1–6 Yamadaoka, Suita, Osaka 565–0871, Japan; 5 iPS Cell-based Research Project on Hepatic Toxicity and Metabolism, Graduate School of Pharmaceutical Sciences, Osaka University, 1–6 Yamadaoka, Suita, Osaka 565–0871, Japan; 6 The Center for Advanced Medical Engineering and Informatics, Osaka University, 1–6, Yamadaoka, Suita, Osaka, 565–0871, Japan; University of Kansas Medical Center, UNITED STATES

## Abstract

The blood brain barrier (BBB) is formed by brain microvascular endothelial cells (BMECs) and tightly regulates the transport of molecules from blood to neural tissues. *In vitro* BBB models from human pluripotent stem cell (PSCs)-derived BMECs would be useful not only for the research on the BBB development and function but also for drug-screening for neurological diseases. However, little is known about the differentiation of human PSCs to BMECs. In the present study, human induced PSCs (iPSCs) were differentiated into endothelial cells (ECs), and further maturated to BMECs. Interestingly, C6 rat glioma cell-conditioned medium (C6CM), in addition to C6 co-culture, induced the differentiation of human iPSC-derived ECs (iPS-ECs) to BMEC-like cells, increase in the trans-endothelial electrical resistance, decreased in the dextran transport and up-regulation of gene expression of tight junction molecules in human iPS-ECs. Moreover, Wnt inhibitors attenuated the effects of C6CM. In summary, we have established a simple protocol of the generation of BMEC-like cells from human iPSCs, and have demonstrated that differentiation of iPS-ECs to BMEC-like cells is induced by C6CM-derived signals, including canonical Wnt signals.

## Introduction

The blood brain barrier (BBB), which is formed by specialized brain microvascular endothelial cells (BMECs) working together with astrocytes and pericytes, plays important roles in brain homeostasis and neuronal functions by regulating the transit of substances from peripheral circulation to brain. Unlike endothelial cells (ECs) that reside in the other tissues or organs, BMECs highly express genes associated with tight junction molecules and efflux/influx transporters, and thereby could regulate the entrance of various types of compounds such as small molecules and drugs, into the brain [1]. To analyze the function of BBB and to examine the permeability of compounds through the BBB, *in vitro* BBB models have been developed using cultured BMECs that were mostly derived from non-human animals [1,2]. However, since the expression pattern and the expression levels of transporters are different between non-human animals and human [3,4], establishment of *in vitro* BBB models using human BMECs would be preferable. Nonetheless, the use of human primary BMECs also has some drawbacks. One is their limited range of sources and differences in function from batch to batch. In addition, although immortalized human BMECs have been established by the transduction of tumor genes including SV40 large T-antigen, these cells show lower barrier functions compared to primary BMECs in general[5,6].

Human pluripotent stem cells (PSCs), such as embryonic stem cells [7,8] and induced PSCs (iPSCs) [9,10], can differentiate into various types of cells in the body in an unlimited quantity. Human PSC-derived ECs are therefore expected to be used as sources for human BMECs. Many researchers have reported the differentiation of human PSCs into ECs using various strategies [11–14]. However, few studies investigated the generation of tissue-specific ECs, including BMECs, from human PSCs.

Since BBB is formed simultaneously with brain development, it was reasonably assumed that BMECs would be maturated by stimulation of the factors produced by other types of cells including neural tissue-related cells. In this study, we initially attempted to establish a method for the differentiation of human iPSCs into ECs under serum- and feeder-free conditions, and examined whether human iPSC-derived ECs (iPS-ECs) could be further maturated to BMEC-like cells by co-culture with several kinds of cultured cell lines. In addition, the effects of cell line-derived conditioned medium on the differentiation of iPS-ECs into BMEC-like cells were also examined to establish a simple protocol for the generation of BMEC-like cells from human iPSCs.

## Results

### Differentiation of ECs from human iPSCs under a serum-free condition

**Fig 1:**
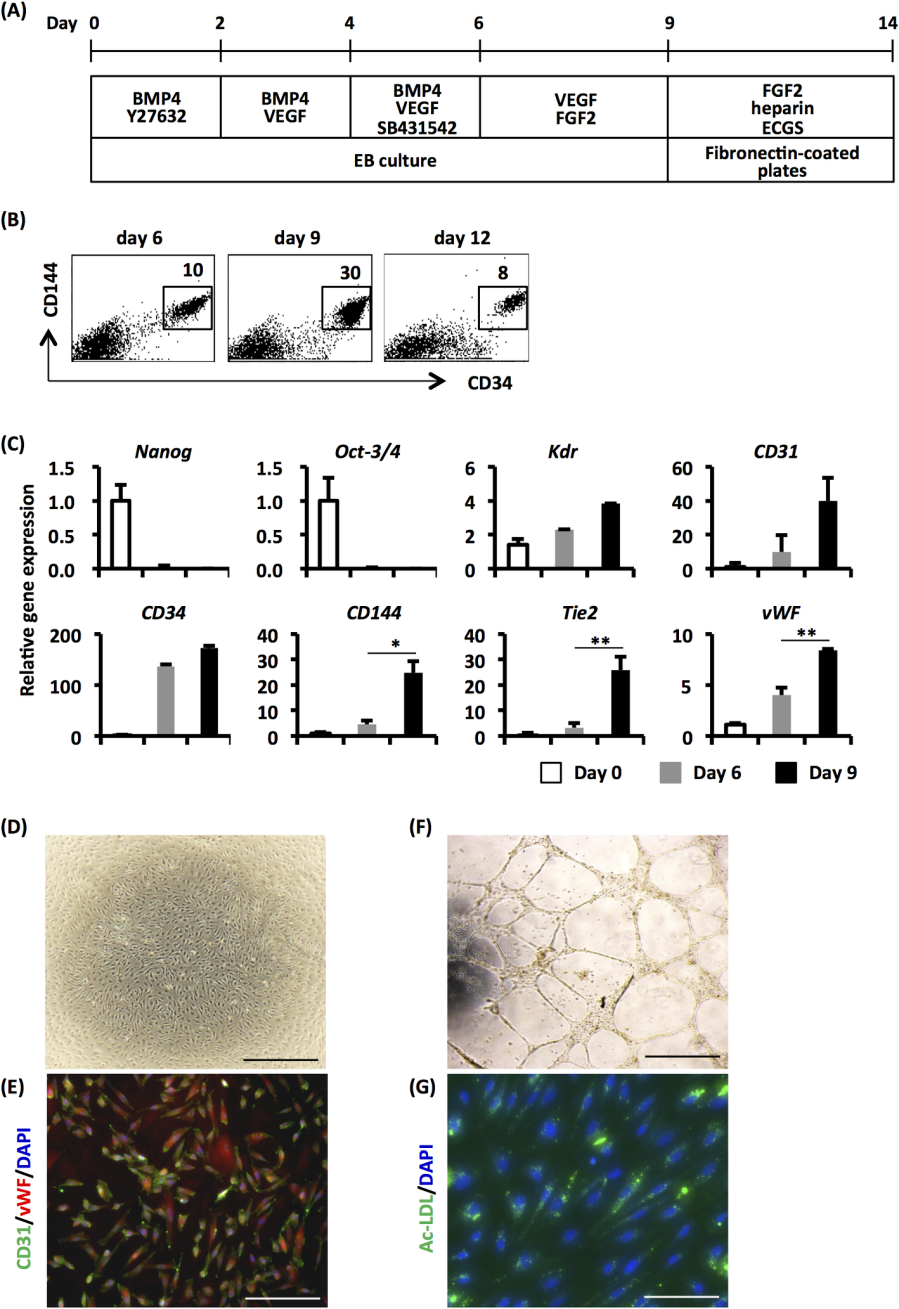
Differentiation of human iPSCs into ECs. (A) EC differentiation of human iPSCs. The detailed differentiation procedure is described in the Materials and Methods section. (B) Human iPSC-derived EBs (day 6, day 9 and day 12) were stained with anti-CD34 and anti-CD144 antibodies, and were then subjected to flow cytometric analysis. Representative results from one of the three independent experiments are shown. (C) Total RNA was extracted from undifferentiated human iPSCs (day 0) and human iPSC-derived EBs (day 6 and day 9). Then, qRT-PCR analysis was performed. Results shown are the mean of three independent experiments with the indicated standard deviations (S.D.). * p < 0.05, ** p < 0.01. (D-G) Sorted CD34^+^ cells were cultured with FGF2, ECGS, and heparin on fibronectin-coated plates. They showed an endothelial-like morphology under these culture conditions (D). These cells were stained positive for CD31 and vWF (E). They were also capable of forming tube-like structures on Matrigel (F) and demonstrated acetylated-LDL uptake (G). The scale bar indicates 300 μm (D) or 100 μm (E, F, G).

To generate brain-specific ECs from human iPSCs, human iPSCs were initially differentiated into ECs under serum-free conditions as illustrated in Fig 1A. Flow cytometric analysis revealed that the degree of CD34^+^CD144^+^ endothelial progenitor cells in EB cells was increased to a peak on day 9, and decreased over the next 3 days (Fig 1B). We also observed the enhanced expression of EC-related genes in EBs on day 9 compared to those in EBs on day 6 (Fig 1C). The expression of PSC-marker genes, Nanog and Oct-3/4, was markedly decreased following EB cultures (Fig 1C). Thus, under our culture conditions, a large number of endothelial lineage cells were observed in 9-day-cultured EBs. To obtain pure ECs, we isolated CD34^+^CD144^+^ cells using anti-human CD34 antibodies, because all CD144^+^ cells highly expressed CD34 (Fig 1B). After cell sorting, EC-like cells were observed from CD34^+^ cell fractions in fibronectin-coated plate culture (Fig 1D), and these cells expressed CD31 and vWF (Fig 1E). Moreover, in these cells, a vascular-like structure was formed in Matrigel (Fig 1F) and uptake of the acetylated LDL was observed (Fig 1G). By contrast, CD34^-^ cell-derived cells did not express CD31 and vWF, and did not show tube formation potential (S1 Fig). Therefore, these results clearly demonstrated that functional ECs were generated from human iPSCs under serum- and feeder-free conditions.

### Induction of BMEC-like cells from human iPS-ECs by co-culture with C6 cells

Recent studies have indicated the pivotal roles of tissue microenvironments in the acquisition of tissue-specific properties of ECs [15]. In addition, iPS-ECs were considered to be “naïve” or “uncommitted” ECs, probably because they were generated and expanded in the absence of tissue-specific factors. Thus, it is expected that co-culture of “naïve” iPS-ECs with neural tissue-related cell lines would lead to the generation of BMEC-like cells with tight junction-forming potential. We screened cultured cell lines that have activities potentiating the barrier function of ECs using b.End3 cells (a mouse BMEC line), and found that rat C6 glioma cells have the strongest capacity to promote the tight junction formation of b.End3 cells (S2 Fig). Thus, BMEC-like cells were generated from iPS-ECs by co-culture with C6 cells. HUVECs were also co-cultured with C6 cells as a control.

**Fig 2:**
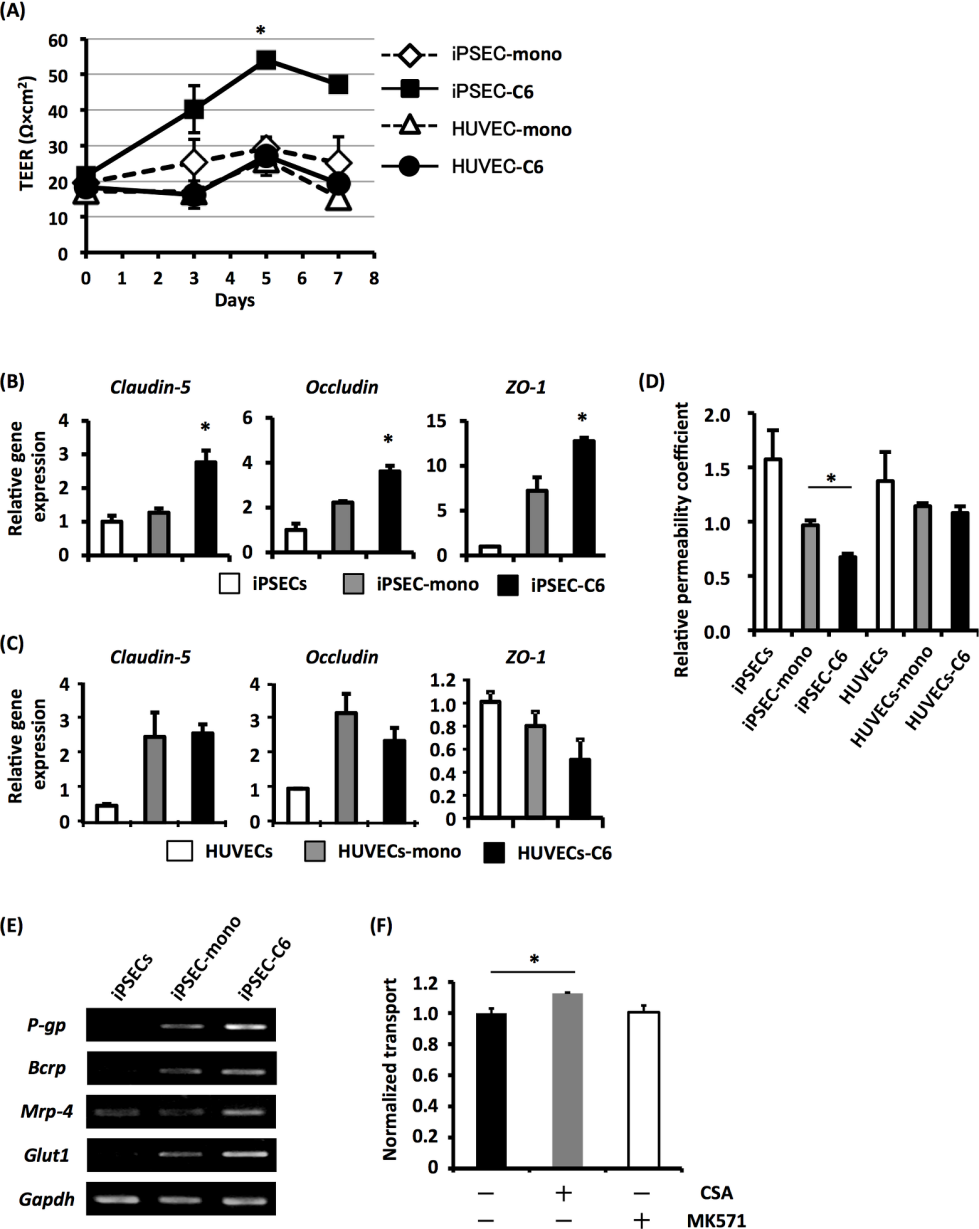
Induction of BMEC-like properties in hiPS-ECs by co-culture with C6 cells. (A) hiPS-ECs and HUVECs were cultured on fibronectin-coated inserts. When confluent, these inserts were transferred onto non-cultured 24-well plates (iPSEC-mono or HUVEC-mono) or C6 cell-cultured 24-well plates (iPSEC-C6 or HUVEC-C6). Then, TEER values for hiPS-ECs and HUVECs were measured at indicated days. (B, C) Expressions of tight junction-related genes (*Claudin-5*, *Occludin* and *ZO-1*) were examined by qRT-PCR analysis before (iPSECs, HUVECs) and after (iPSEC-mono, iPSEC-C6, HUVEC-mono, HUVEC-C6) 5 days of culture. (D) The permeability coefficient for FD was investigated in 5-day co-cultured hiPS-ECs and HUVECs. (E) Expressions of transporter genes (*P-gp*, *Bcrp*, *Mrp-4* and *Glut1*) were examined in 5-day mono- or co-cultured hiPS-ECs by RT-PCR analysis. (F) The 5-day co-cultured hiPS-ECs were treated with CSA (an inhibitor of P-gp) or MK571 (an inhibitor of MRP-4), and then the permeability coefficient for Rhodamin 123 (a specific substance of P-gp) was measured. All results shown are the mean of three independent experiments with the indicated standard deviations (S.D.). * p < 0.05.

The TEER value of iPS-EC monolayers (iPSEC-mono) was 22–28 Ω cm^2^ and was unchanged after the culture on Transwell inserts, whereas that of iPS-ECs co-cultured with C6 cells (iPSEC-C6) was gradually increased and peaked on day 5 (approximately 55 Ω cm^2^) (Fig 2A). We also observed a significant elevation of gene expression of tight junction-related genes, *Claudin-5*, *Occludin*, and *Zonula occludens-1* (*ZO-1*), in iPSEC-C6 (Fig 2B). Furthermore, co-culture with C6 cells led to a decrease in dextran (MW 3,000) transport in iPS-ECs (Fig 2D). These data thus indicate that iPSEC-C6 could form tight junctions and thereby could have barrier functions. By contrast, the barrier functions of HUVECs were not altered even in the presence of C6 cells (Fig 2A–2D).

To provide nutrients to the brain and to protect the brain from exposure to unnecessary metabolites and drugs, BMECs express various types of influx and efflux transporters. Thus, the expression levels of transporter genes in iPSEC-mono and iPSEC-C6 were investigated by RT-PCR. The results showed that, compared with iPSEC-mono, iPSEC-C6 showed an increased expression of *Mdr1*, *Bcrp*, *Mrp-4* and *Glut1* (Fig 2E). We next examined the function of a transporter p-gp in iPSEC-C6 using rhodamine123, a substrate for p-gp, and cyclosporine A, an inhibitor of p-gp. As shown in Fig 2F, the transport of rhodamine123 from the upper chambers to the lower chambers was slightly but significantly increased by treatment with cyclosporine A, but not by treatment with MK571, an inhibitor of the MRP family, indicating that iPSECs-C6 could express functional p-gp. Together, these results suggest that iPS-ECs would be maturated to BMEC-like cells, which form tight junctions and express various types of transporters, by co-culture with C6 cells.

### Maturation of iPS-ECs to BMEC-like cells using C6 cell-conditioned medium (C6CM)

**Fig 3:**
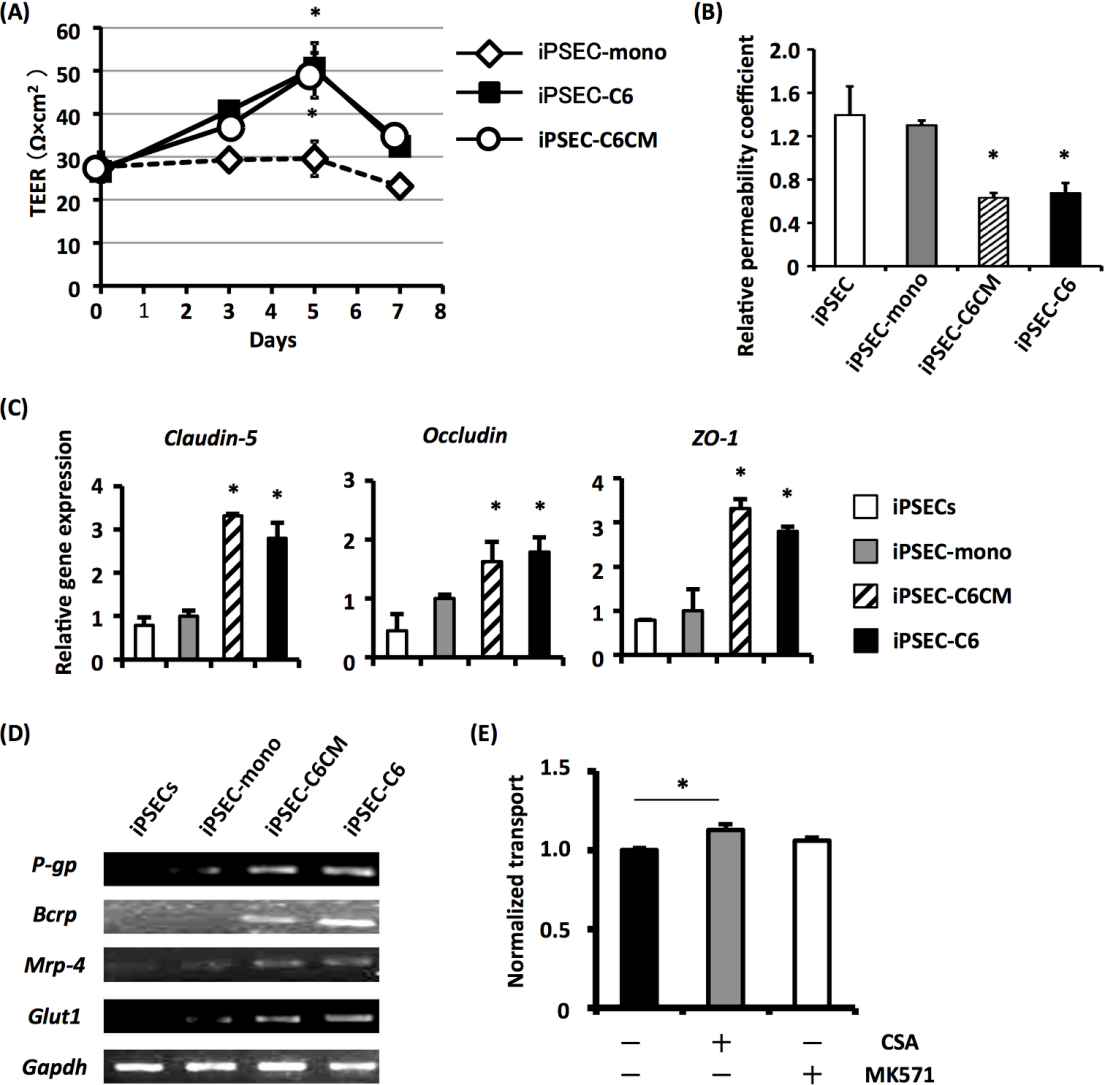
Induction of BMEC-like properties of hiPS-ECs by treatment with C6CM. (A) hiPS-EC monolayers were transferred onto non-cell culture (iPSEC-mono), C6-cell culture (iPSEC-C6) or C6CM (iPSEC-C6CM) in 24-well plates. Then, the TEER value of each hiPS-EC monolayer was measured at indicated days. (B) The permeability coefficient for FD was measured in hiPS-ECs before (iPSECs) and after 5-day mono-culture (iPSEC-mono), 5-day co-culture with C6 cells (iPSEC-C6) or 5-day culture in C6CM (iPSEC-C6CM). (C) Expressions of tight junction-related genes (*Claudin-5*, *Occuludin* and *ZO-1*) were examined in iPSECs, iPSEC-mono, iPSEC-C6 and iPSEC-C6CM. (D) Expressions of transporter genes (*P-gp*, *Bcrp*, *Mrp-4* and *Glut1*) were examined in iPSECs, iPSEC-mono, iPSEC-C6 and iPSEC-C6CM by RT-PCR analysis. (E) The iPSEC-C6 was treated with CSA or MK571, and then the permeability coefficient for Rhodamin 123 was investigated in them. All results shown are the mean of three independent experiments with the indicated standard deviations (S.D.). * p < 0.05.

Considering that C6 cells did not directly contact iPS-ECs under our culture conditions, the results in Fig 2 suggest that C6 cell-derived soluble factors promote the maturation of iPS-ECs to BMEC-like cells. We thus investigated the effect of C6CM on the maturation of iPS-ECs. Interestingly, TEER measurement, gene expression analysis and dextran transport assay showed the increased barrier properties of iPS-ECs in the presence of C6CM (iPSEC-C6CM), as in the case of co-culture with C6 cells (Fig 3A–3C). Furthermore, addition of C6CM to the cultures resulted in the enhanced expression of transporter genes in iPS-ECs (Fig 3D). We also confirmed the functionality of p-gp expressed in iPSECs-C6CM (Fig 3E). Therefore, C6 cell-derived soluble factors would be sufficient to induce maturation of iPS-ECs to BMEC-like cells.

### Involvement of Wnt signaling in the maturation of iPS-ECs to BMEC-like cells

**Fig 4:**
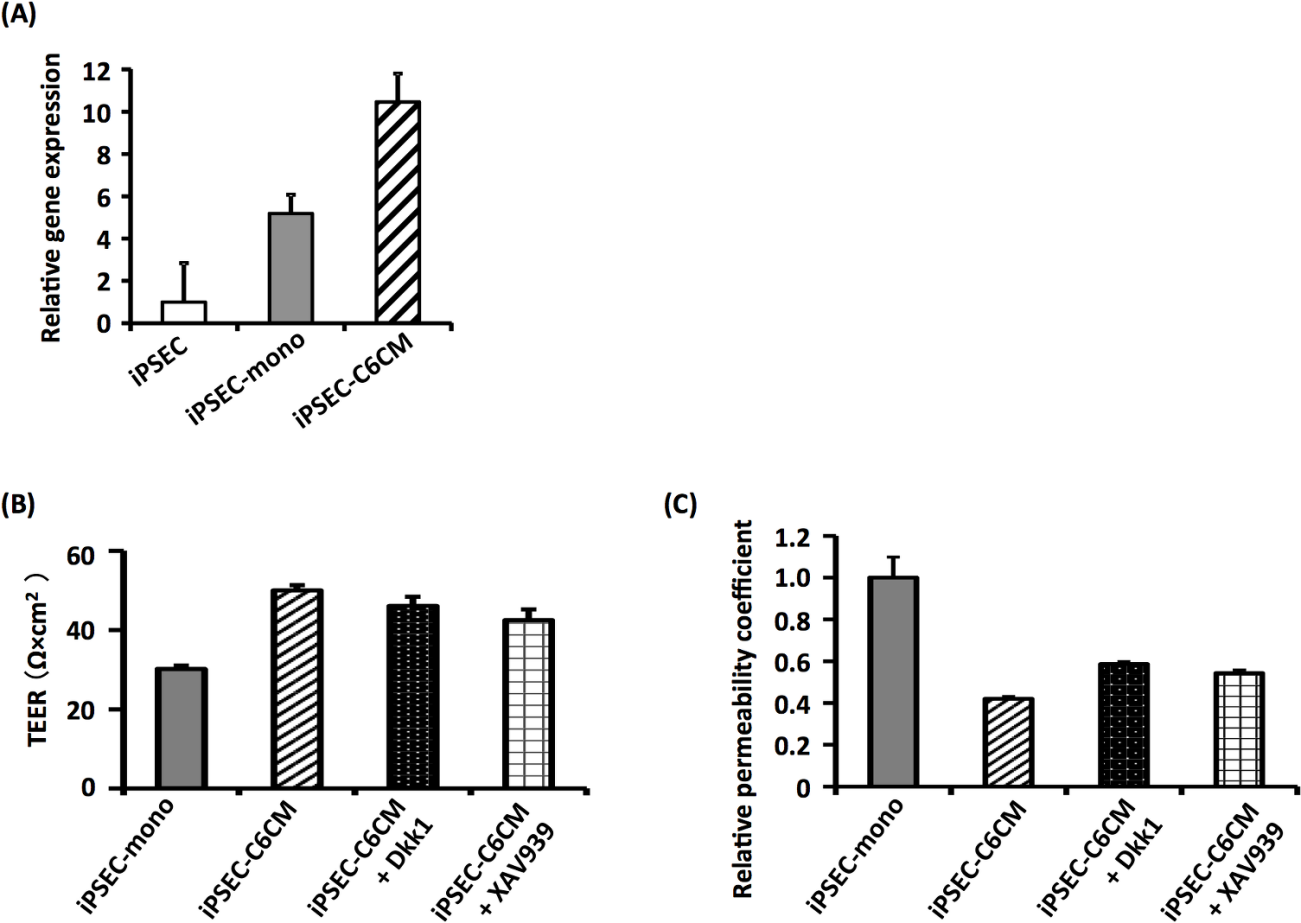
Effects of inhibitors of the canonical Wnt pathway on the differentiation of hiPS-ECs into BMEC-like cells by treatment of C6CM. (A) Expression of *Axin-2* mRNA was examined in iPSECs, iPSEC-mono and iPSEC-C6CM by qRT-PCR analysis. (B, C) TEER values and permeability coefficients of iPSEC-mono, iPSEC-C6CM or Dkk1- or XAV939-treated iPSEC-C6CM were measured. All results shown are the mean of three independent experiments with the indicated standard deviations (S.D.).

Canonical Wnt signaling via β-catenin has been reported to contribute to angiogenesis in the brain [16–18]. Therefore, we expected that C6CM would stimulate the canonical Wnt pathway in iPS-ECs, and the activated Wnt signaling would lead to the maturation of iPS-ECs to BMEC-like cells. Indeed, the expression of Axin2, a target gene of ß-catenin, was increased in iPSEC-C6 (Fig 4A), suggesting activation of the canonical Wnt pathway. We studied the involvement of canonical Wnt signaling in the maturation of iPS-ECs using inhibitors (Dkk1 and XAV931), and found that the inhibition of canonical Wnt signaling by Dkk1 and XAV931 led to a slight decrease in the barrier functions of iPSECs-C6CM, although Dkk1- or XAV931-treated iPSEC-C6CM still displayed barrier functions superior to those of iPSEC-mono (Fig 4B and 4C). Thus, these results suggest that C6 cell-induced maturation of iPS-ECs to BMEC-like cells would be partially mediated by canonical Wnt signaling.

## Discussion

The aim of this study is to generate human BMECs from PSCs, because an establishment of *in vitro* BBB models using human BMECs would be required to analyze the BBB functions and to perform drug-screening for neurodegenerative diseases. In the present study, we initially succeeded in the generation of iPS-ECs under serum- and feeder-free conditions (Fig 1). Furthermore, we showed that the TEER values in iPSEC-C6 were higher than those in iPSEC-mono, HUVEC-mono, and HUVEC-C6 cells, and that paracellular transport was significantly reduced in iPSEC-C6, indicating the increased barrier functions (Fig 2). In addition, we found an elevation of the expression level of genes involved in influx and efflux transporters in iPS-ECs by co-culture with C6 cells (Fig 2). The TEER values (~55 Ω cm^2^) in iPSEC-C6 were largely identical to those in hCMEC/D3 cells (30~40 Ω cm^2^) [5], which are immortalized human BMECs that have been widely utilized as cell sources for human *in vitro* BBB models [19,20]. Our results, therefore, indicate that iPS-ECs would be maturated into BMEC-like cells by co-culture with C6 cells.

C6 cells have the properties of astrocytes and maintain the functionality of primary BMECs *in vitro* [21,22]. It has also been shown to increase in the barrier functions of primary BMECs *in vitro* by treatment of C6CM [23]. In addition to these functions, we found, for the first time, that C6 cells could facilitate the maturation of “naïve” iPS-ECs into BMEC-like cells. On the other hand, C6 cells had almost no impact on the barrier functions and the expression levels of transporter genes in HUVECs. Because HUVECs were derived from “mature” or “committed” ECs in the umbilical cord, they might not be converted into BMEC-like cells even in the presence of C6 cell-derived factors. These findings suggest the possibility that both “naïve” ECs and signals derived from neural tissue-related cells would be necessary to induce the BMEC-like cells. Considering the difficulty in obtaining a large amount of human primary BMECs and the difficulty of conversion of tissue- or organ-derived mature ECs into BMECs, iPS-ECs would be an ideal cell source for human BMEC generation. In the future, therefore, it will be important to investigate whether ECs derived from other kinds of iPSCs could differentiate into BMEC-like cells by co-culture with C6 cells.

Another important finding in this study was that C6CM could also promote the maturation of iPS-ECs into BMEC-like cells. These data suggest that C6 cell-derived soluble factors would play an important role in the maturation of iPS-ECs. Consistent with the previous findings, we confirmed that canonical Wnt signaling contributed to the maturation of iPS-ECs by C6CM treatment. However, not only the canonical Wnt pathway but also other unknown factors are likely to be involved in the C6CM-elicited maturation of iPS-ECs, since treatment of canonical Wnt inhibitors did not completely block the maturation of iPS-ECs. The identification of C6 cell-derived maturation factors in the C6CM would be important to elucidate the maturation mechanisms induced by C6 cells.

Recently, Lippmann et al. showed that BMEC-like cells could be generated from human PSCs by spontaneous differentiation cultures [24,25]. BMEC-like cells generated by their methods had potent barrier functions, but the differentiation efficiencies were assumed to be unstable, due to the dependence on random differentiation. On the other hand, we generated BMEC-like cells from human iPSCs using strategies different from Lippmann’s. It would be of interest to compare the characteristics of BMEC-like cells generated from the different protocols.

In summary, we showed that iPS-ECs could be differentiated into BMEC-like cells by co-culture with C6 cells or by treatment with C6CM. Maturation levels of BMEC-like cells generated in this study, however, are still insufficient because the TEER values and the expression levels of transporter genes in iPS cell-derived BMEC-like cells are low in comparison with those in primary BMECs [2]. Co-culture of iPS-ECs with human neural tissue-related cells, instead of non-human cell lines, might possibly be effective for generation of fully maturated BMECs from human PSCs. Although further study will be needed to induce the fully maturated BMECs, human iPSC-derived BMECs would be a potential cell source for the generation of *in vitro* BBB models, and are also expected to be of importance in drug-screening.

## Materials and Methods

### Cell culture

The human iPSC line, 201B7 (provided by Dr. S. Yamanaka, Kyoto University) [9], was maintained on mitomycin C-treated mouse embryonic fibroblasts (Merk-Millipore, Billerica, MA) with Repro Stem medium (ReproCELL, Yokohama, Japan) supplemented with 5 ng/ml human fibroblast growth factor 2 (FGF2: Katayama Kagaku Kogyo, Osaka, Japan). Human umbilical vein endothelial cells (HUVECs) were purchased from Lonza (Walkersville, MD) and cultured in EGM-2 medium (Lonza). b.End3 (an immortalized mouse BMEC line) cells were obtained from American Type Culture Collection, and were cultured in Dulbecco’s modified Eagle’s medium (DMEM: Wako, Osaka, Japan) (high-glucose) supplemented with sodium pyruvate, 10% fetal bovine serum (FBS: Life Technologies, Carlsbad, CA), and antibiotics (120 μg/ml streptomycin and 200 μg/ml penicillin, Life Technologies). A1 (a mouse astrocyte-like cell line), MG-5 (a mouse microglial cell line), and C6 (a rat glioma cell line) were provided by the JCRB cell bank (Osaka, Japan). A1 cells were cultured in DMEM (high-glucose) supplemented with 10% FBS and antibiotics. MG-5 cells were cultured in a 3:7 mixture of DMEM (high-glucose) supplemented with 10% FBS and A1 cell-conditioned medium (CM). C6 cells were cultured in Ham’s F10 (Life Technologies) supplemented with 15% horse serum (Nichirei, Tokyo, Japan), 2.5% FBS, and antibiotics. RAW 264.7 and J774.1 (mouse macrophage-like cell lines) cells were cultured in DMEM (low-glucose) (Sigma, St. Louis, MO) and RPMI1640 (Sigma), respectively, supplemented with 10% FBS and antibiotics.

### EC differentiation

Human iPSCs were dissociated into single cells with Accutase (Merk-Millipore). The cells were re-suspended in differentiation medium (StemPro-34 medium (Life Technologies) containing StemPro-34 Nutrient Supplement (Life Technologies), 50 μg/ml ascorbic acid (Sigma), 0.45 mM 1-thioglycerol (MTG: Sigma), 2 mM L-glutamine (Life Technologies), and antibiotics) supplemented with 20 ng/ml human bone morphogenetic protein 4 (BMP4: R&D Systems, Mineapolis, MN), 2 ng/ml Activin A (R&D Systems), and 10 μM Y27632 (a ROCK inhibitor, Wako), and were then plated on a Lipidure-coated round-bottom 96-well plate (Thermo Scientific, Waltham, MA) at 2×10^4^ cells per well to form embryoid bodies (EBs) (day 0). On day 2, EBs were cultured in differentiation medium supplemented with 20 ng/ml human BMP4 and 5 ng/ml human vascular endothelial growth factor (VEGF: Peprotech, Rocky Hill, NJ). On day 4, the medium was changed to differentiation medium supplemented with 20 ng/mL human BMP4, 5 ng/mL human VEGF, and 10 μM SB431542 (an inhibitor of TGFß/Activin/Nodal pathway, Wako). On day 6, EBs were harvested and resuspended in differentiation medium supplemented with 20 ng/ml human VEGF and 5 ng/ml FGF2 before plating on a 10 cm Petri dish. Then, EB-derived cells were sorted by FACS. FACS-sorted cells were cultured in EC medium (differentiation medium supplemented with 100 μg/ml endothelial cell growth supplement (ECGS, Sigma), 100 μg/ml heparin (Sigma), and 20 ng/ml FGF2) on fibronectin (20 μg/cm^2^: BD Bioscience, San Jose, CA)-coated plates or Transwell permeable inserts (0.4 μm pore size: BD Bioscience). The medium was changed every 2 days until the cells were used for further experiments.

### Flow cytometry and cell sorting

Cells (3×10^4^ to 1×10^5^) were incubated with an APC-conjugated anti-human CD34 antibody (clone: 581, Biolegend San Diego, CA) and a PE-conjugated anti-human VE-Cadherin/CD144 antibody (clone: 16B1, e-Bioscience, San Diego, CA) at 4°C for 40 min and washed twice with staining buffer (PBS/2% FBS). Analysis was performed on an LSRFortessa flow cytometer by using FACSDiva software (BD Bioscience). For cell sorting, human iPSC-derived EBs were dissociated using 0.25% trypsin/EDTA (Life Technologies) and were then stained with an APC-conjugated anti-human CD34 antibody. CD34-expressing cells were isolated by an SH-800 cell sorter (Sony). Dead cells were excluded from the analysis by staining with Hoechst33342 (1 μg/mL, Life Technologies).

### RT-PCR

Total RNA was isolated using ISOGEN (Nippon Gene, Tokyo, Japan) or RNAiso Plus (TaKaRa, Shiga, Japan). cDNA was synthesized using a SuperScript VILO cDNA synthesis kit (Life Technologies), and semi-quantitative PCR was then performed using TaKaRa ExTaq HS DNA polymerase (Takara). Semi-quantitative PCR reactions were performed by heating to 94°C for 2 min, and then the samples were subjected to number of cycles of 94°C for 15 sec, 55°C for 30 sec with 72°C for 30 sec and a final extension of 72°C for 1 min. In the case of C6 cell-derived cDNA, 3% dimethyl sulfoxide was added. The product was analyzed by 2% agarose/TBE gel electrophoresis followed by staining with ethidium bromide. Quantitative real-time RT-PCR was performed with Fast SYBR Green Master Mix using an ABI StepOne Plus system (Life Technologies). Relative quantification was performed against a standard curve and the values were normalized against the input determined for the housekeeping gene, glyceraldehyde 3-phosphate dehydrogenase (GAPDH). The sequences of the primers used in this study are listed in Tables 1 and 2.

**Table 1:** Primer list used in Semi-quantitative PCR.

Gene Name	(5') Forward Primers (3')	(5') Reverse Primers (3')
*Gapdh*	ACCACAGTCCATGCCATCAC	TCCACCACCCTGTTGCTGTA
*Mdr1*	GCCTGGCAGCTGGAAGACAAATACACAAAATT	CAGACAGCAGCTGACAGTCCAAGAACAGGACT
*Bcrp*	TTATCCGTGGTGTGTCTGGA	CCTGCTTGGAAGGCTCTATG
*Mrp4*	CCATTGAAGATCTTCCTGG	GGTGTTCAATCTGTGTGC
*Glut-1*	CCTGCAGGAGATGAAGGAAG	TGAAGAGTTCAGCCACGATG

**Table 2:** Primer list used in quantitative real-time PCR.

Gene Name	(5') Forward Primers (3')	(5') Reverse Primers (3')
*Gapdh*	GGTGGTCTCCTCTGACTTCAACA	GTGGTCGTTGAGGGCAATG
*Nanog*	AGAAGGCCTCAGCACCTAC	GGCCTGATTGTTCCAGGATT
*Oct-3/4*	CTTGAATCCCGAATGGAAAGGG	GTGTATATCCCAGGGTGATCCTC
*Kdr*	ACTTTGGAAGACAGAACCAAATTATCTC	TGGGCACCATTCCACCA
*CD31*	GAGTATTACTGCACAGCCTTCA	AACCACTGCAATAAGTCCTTTC
*CD34*	CTACAACACCTAGTACCCTTGGA	GGTGAACACTGTGCTGATTACA
*CD144*	TCACGATAACACGGCCAACA	TGGCATCCCATTGTCTGAGA
*Tie2*	GCTAGAGTCAACACCAAGGCC	TCCAAGAAATCACAGCTGAGGA
*vWF*	AGTGCAGACCCAACTTCACC	GTGGGGACACTCTTTTGCAC
*Claudin-5*	CTTCCAGAATGGCAAGAGAGTGA	ACCACTGTTCTCCACTGCTCAGA
*Occludin*	ACAAGCGGTTTTATCCAGAGTC	GTCATCCACAGGCGAAGTTAAT
*Zo-1*	TGATCATTCCAGGCACTCG	CTCTTCATCTCTACTCCGGAGACT
*Axin2*	GAGTGGACTTGTGCCGACTTCA	GGTGGCTGGTGCAAAGACATAG

### Immunostaining

Cells were washed twice with PBS and fixed in 4% paraformaldehyde (PFA: Wako) at room temperature for 10 min. After washing with PBS, cells were permeabilized with PBS containing 0.1% Triton X-100 for 10 min, blocked with PBS containing 2% bovine serum albumin for 20 min, and incubated with an antibody against CD31 (clone: JC70A, Dako) and von Willebrand factor (vWF: Cat No. A0082, Dako) at 4°C overnight. Then, cells were incubated with AlexaFluor488- or Alexafluor594-conjugated secondary antibodies (1:1000; Life Technology) at room temperature for 1 hr. After washing, nuclei were labeled with 4’, 6-diamidino-2-phenylindol (DAPI: Sigma).

### Tube-forming assay and acetylated low-density lipoprotein (LDL) uptake assay

Plates (48-wells) were coated with 100 μl of Matrigel Matrix (BD Bioscience) at 37°C for 1 hr. FACS-sorted cells, which were cultured on fibronectin-coated plates as described above, were harvested using 0.25% trypsin/EDTA, and were then seeded onto Matrigel-coated plates in differentiation medium supplemented with 10 ng/ml VEGF. Cells were observed by microscopy after incubation for 16 hr. For acetylated LDL uptake assay, cells were incubated with 10 μg/ml acetylated LDL conjugated with AlexaFluor488 (Life Technologies) at 37°C for 4 hr. After incubation, the cells were fixed in ice-cold 4% PFA for 10 min, visualized and photographed under a fluorescent microscope (BIOREVO BZ-9000, Keyence, Osaka, Japan).

### Effects of C6 co-culture and C6CM on differentiation

Human iPSC-derived CD34^+^ cells were plated and cultured on fibronectin-coated Transwell inserts (24 well plates) in differentiation medium as described above. When confluent, human iPS-EC monolayers were transferred onto sub-confluent C6 cells in 24-well plates. Then, the cells were further cultured in the EC medium (upper chamber) and differentiation medium (lower chamber). To prepare C6CM, C6 cells were cultured on 10 cm dishes until confluent, and then the medium was replaced with 8 ml of differentiation medium. After 24 hr of incubation, the medium was collected, filtered, and stored at -80°C. When C6CM was used, human iPS-EC monolayers, which were cultured on Transwell inserts until confluent, were transferred onto 24-well plates, and EC medium was loaded onto upper chambers. We added the C6CM (100%) to the lower chambers to mimic the co-culture experiments with C6 cells. In some experiments, 200 ng/ml human Dickkopf1 (Dkk1: Peprotech) or 10 μM XAV939 (Sigma) was added to the cultures. After cells were cultured for the indicated number of days, the characteristics of human iPS-ECs were studied, as described below.

### Measurement of trans-endothelial electrical resistance (TEER)

TEER values of human iPS-EC monolayers or HUVEC monolayers on Transwell inserts (24-well plates) were measured using Millicell ERS-2 (Merk-Millipore). To calculate TEER (Ω (0 ohrms)×cm^2^ (the surface area of the insert, 0.3 cm^2^)), the resistance of the fibronectin-coated insert without cells (blank resistance) was always subtracted from the resistance of cell cultures (sample resistance) [2,5]

### Permeability experiments

Human iPS-ECs and HUVECs were cultured on Transwell inserts for 5 days. Before the transport studies, the medium was removed from the upper and lower chambers and the chambers were washed with EBM basal medium phenol red free (Lonza) containing 0.1% FBS (0.1% FBS/EBM). Medium containing 100 μg/ml fluorescein-conjugated dextran (FD, MW 3,000: Life Technologies) was then loaded onto the upper chambers, and 0.1% FBS/EBM was added to the lower chambers. After 15 hr of incubation, the medium in the lower chamber was collected. The concentration of FD in the sample was determined using a fluorescence multiwell plate reader (Ex(λ) 494 nm, Em(λ) 521 nm: Genios, TECAN, Männedorf, Switzerland). The permeability coefficient was calculated as previously reported [26,27].

### P-glycoprotein (P-gp) functionality assays

P-gp functionality was assessed using rhodamine 123 (Sigma), a substrate for P-gp, and efflux transporter inhibitors as follows. Human iPS-ECs, which were co-cultured with C6 cells or incubated in C6CM, were washed with PBS and pre-incubated at 37°C for 1 hr with or without inhibitors (5 μM cyclosporine A (Wako) or 10 μM MK571 (Sigma)), followed by addition of rhodamine 123 to the upper chamber. After 1 hr, the fluorescence activity of the medium in the lower chamber was quantified on a plate reader [24].

### Statistical analysis

Statistical analysis was performed using unpaired two-tailed Student’s t-test.

## Supporting Information

S1 Fig Endothelial characterization of hiPSC-derived CD34^-^ cells. Sorted hiPSC-derived CD34^-^ cells were cultured with FGF2, ECGS and heparin on fibronectin-coated plates. (A) Expressions of CD31 and vWF were undetectable in these CD34^—^derived cells. (B) The acetylated-LDL uptake could not be observed. (C) The tube-like structure formation on Matrigel was not detectable. The scale bar indicates 100 μm (A, B) or 300 μm (C).
(PDF)

S2 Fig TEER values for b.End3 cells co-cultured with cell lines. b.End3 cells were co-cultured with A1 cells (astrocytic cell line), C6 cells (glioma cell line), MG5 cells (microglial cell line), RAW264.7 cells and J774.1 cells (macrophage cell lines) for 6 days. The TEER value of each b.End3 cell monolayer was measured.
(PDF)
