# Supplementary figures and images for: Generation of Brain Microvascular Endothelial-Like Cells from Human Induced Pluripotent Stem Cells by Co-Culture with C6 Glioma Cells
Source: PLoS One. 2015 Jun 10;10(6):e0128890. doi: 10.1371/journal.pone.0128890 (PMC4464886; doi:10.1371/journal.pone.0128890)

## Supplemental Figure 1 Minami H et al.

(A) CD31/vWF/DAPI

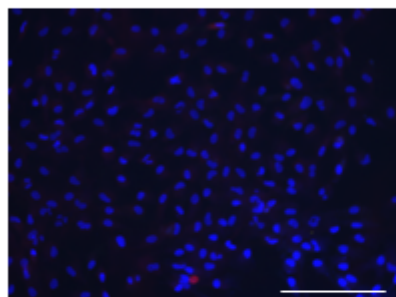

(B) AcLDL/DAPI

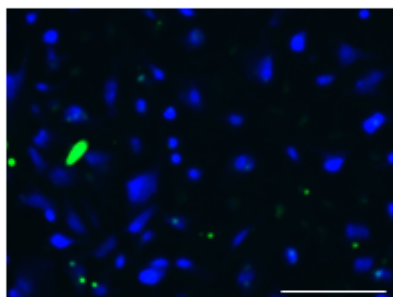

(C)

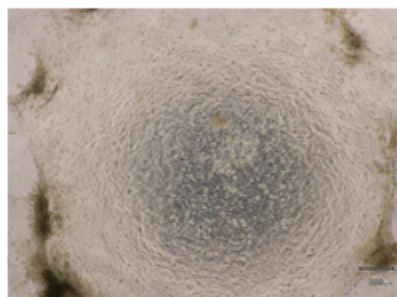

Supplement: S1 Fig — Sorted hiPSC-derived CD34- cells were cultured with FGF2, ECGS and heparin on fibronectin-coated plates. (A) Expressions of CD31 and vWF were undetectable in these CD34—derived cells. (B) The acetylated-LDL uptake could not be observed. (C) The tube-like structure formation on Matrigel was not detectable. The scale bar indicates 100 μm (A, B) or 300 μm (C). (PDF) [file pone.0128890.s001.pdf]

Supplemental Figure 2 Minami H et al.

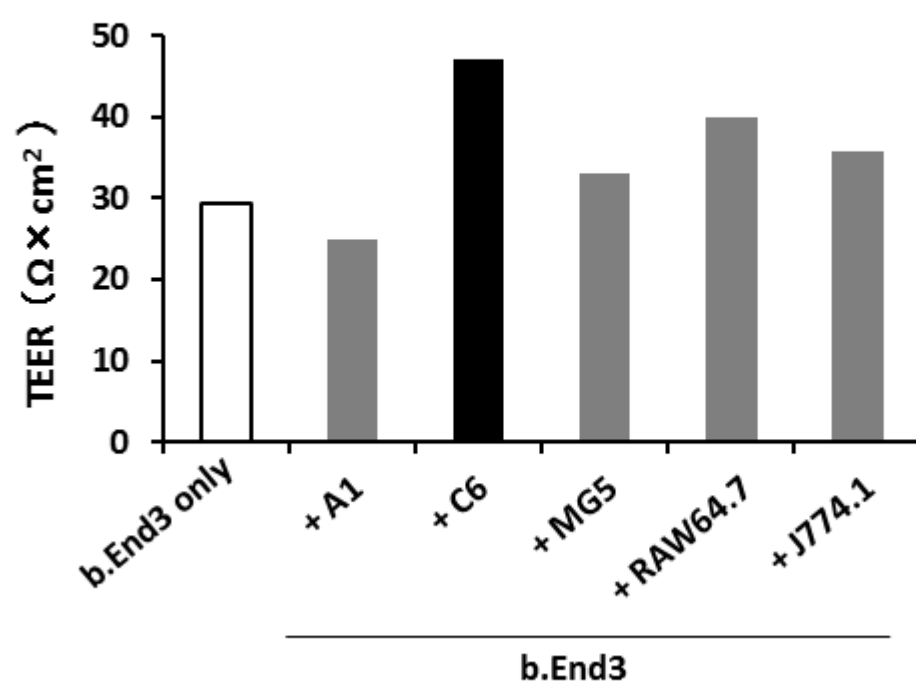

Supplement: S2 Fig — b.End3 cells were co-cultured with A1 cells (astrocytic cell line), C6 cells (glioma cell line), MG5 cells (microglial cell line), RAW264.7 cells and J774.1 cells (macrophage cell lines) for 6 days. The TEER value of each b.End3 cell monolayer was measured. (PDF) [file pone.0128890.s002.pdf]
